# Impact of Psychiatry Clinical Internship on Medical Students’ Specialty Choice

**DOI:** 10.1192/j.eurpsy.2025.2372

**Published:** 2025-08-26

**Authors:** S. Ben Aissa, R. Khouloud, N. Chaima, L. Amine, M. Wahid

**Affiliations:** 1 Psychiatry D, Razi Hospital, Mannouba, Tunisia

## Abstract

**Introduction:**

Medical students often harbor entrenched and somewhat negative views towards psychiatry, which can impact their interest in pursuing this specialty and pose challenges to recruitment efforts.

**Objectives:**

To assess whether exposure to clinical placements in psychiatry can positively shift medical students’ perceptions and influence their career decisions towards this field.

**Methods:**

This study employed a “before/after” longitudinal design over an eight-month period involving 5th-year medical students who were starting their 4-week psychiatric rotation. Evaluations were carried out at the beginning and end of the internship using a comprehensive questionnaire that covered sociodemographic details, clinical exposure, and students’ career inclinations towards psychiatry. The questionnaire also explored various reasons influencing their specialty choice, aiming to identify shifts in perspective directly attributable to their internship experience.

**Results:**

A total of 41 students (n=41) participated in the study. There was a notable positive shift in attitudes towards psychiatry observed at the conclusion of the internship. At the start of the rotation, only 29% of students considered choosing psychiatry as a potential career path. By the end of the internship, this figure had increased to 49%. This substantial increase underscores the transformative potential of direct, immersive experiences in altering career considerations among medical students.

**Conclusions:**

Consistent with the literature, the results of this study demonstrate the positive impact that clinical placements in psychiatry can have on medical students’ attitudes towards the specialty and their subsequent choice of it as a career path. This improvement highlights the critical role that such internships play not only in enhancing educational outcomes but also in shaping the future workforce in psychiatry. It is evident that integrating robust psychiatric placements into medical training programs is essential for nurturing an informed, motivated, and committed next generation of psychiatrists. Our findings support the continued advocacy for and expansion of these experiential learning opportunities within medical curricula to foster a deeper understanding and appreciation of psychiatry.

**Disclosure of Interest:**

None Declared

